# Mycophenolate mofetil and FK506 have different effects on kidney allograft fibrosis in rats that underwent chronic allograft nephropathy

**DOI:** 10.1186/1471-2369-13-53

**Published:** 2012-07-02

**Authors:** Lei Luo, Zhaolin Sun, Weidong Wu, Guangheng Luo

**Affiliations:** 1 Department of Research and Education, Guizhou Province People’s Hospital, Guiyang, China; 2 Department of Urology Surgery, Guizhou Province People’s Hospital, Guiyang, China

**Keywords:** Tacrolimus, Mycophenolate Mofetil, Renal fibrosis, Chronic allograft nephropathy, Kidney transplantation

## Abstract

**Background:**

Tacrolimus (FK506) is associated with renal fibrosis in long-term use. Mycophenolatemofetil (MMF) can also inhibit or attenuate the progression of renal fibrosis. This study aimed to determine the different effects of FK506 and MMF on fibrosis-associated genes in the kidney in rats that underwent chronic allograft nephropathy (CAN).

**Methods:**

Fisher (F344) kidneys were orthotopically transplanted into Lewis rat recipients. All recipients were given Cyclosporin A (CsA) 10 mg/kg^-1^.d^-1^ × 10 day and were then randomly divided into three oral treatment groups (n = 9 in each group): (1) the vehicle group was given vehicle orally; (2) the FK506 group was given 0.15 mg/kg^-1^.d^-1^ FK506; and (3) the MMF group was given 20 mg/kg^-1^.d^-1^ MMF. At 4, 8, and 12 weeks post-transplantation, serum creatinine (SCr), collagen deposition, Connective tissue growth factor (CTGF), alpha smooth muscle actin (*α*-SMA) and E-cadherin expressions were determined and hematoxylin-eosin (HE) and Periodic acid-Schiff (PAS) stains were performed.

**Results:**

Renal function progressively deteriorated and showed typical CAN morphology in the vehicle and FK506 groups, while SCr and inflammatory infiltration (Banff score) showed a significant decrease in the MMF group after 8 weeks post-transplantation compared with those in the other groups (*p* < 0.05). Furthermore, expression levels of CTGF and *α*-SMA in the MMF group were significantly reduced, and the down-regulated expression of E-cadherin was abated (*p* < 0.05).

**Conclusions:**

MMF showed favorable effects on renal interstitial fibrosis, thus efficiently retarding the progression of CAN.

## Background

Despite improvements in immunosuppressive protocols, long-term survival of allografts remains a major impediment [1]. Research has demonstrated that more than 50 % of all renal transplants have graft failure, ultimately due to end-stage renal failure [2]. This process of renal failure was formerly known as chronic allograft nephropathy (CAN), which was originally coined as a histological grading of the extent of interstitial fibrosis (IF) and tubular atrophy (TA) present in biopsies in 1991. Moreover, other histological characteristics can also be involved in CAN, including transplant vasculopathy and glomerulopathy [3,4]. A significant amount of CAN histological damage can appear from as early as a median of 3 months post-transplant, and the condition is progressive [5]. The incremental IF/TA from immunological and non-immunological causes eventually leads to chronic graft dysfunction [4].

A number of short- and long-term animal and human studies on kidney allografts have demonstrated the nephrotoxicity of tacrolimus [6,7]. Reduced dosing or withdrawal of tacrolimus, or addition of mycophenolatemofetil (MMF) in patients generally results in less progression in chronic allograft nephropathy (CAN) indices [8]. MMF is the most commonly used drug for maintenance in transplant patients. By decreasing the intensity of rejection, MMF may prevent subsequent fibrosis and development of CAN [9]. In our previous study, we observed that FK506 up-regulated the expression of TGF-β_1_ and Smad2 in grafts, and down-regulated the expression of Smad7, while MMF had opposite effects [10]. Moreover, it has been reported that MMF can ameliorate transplant fibrosis in an experimental animal CAN model [10,11].

Connective tissue growth factor (CTGF) is a matricellular protein, which plays an important role in pathological fibrosis [12]. Enhanced expression of CTGF is associated with the process of epithelial-mesenchymal transformation (EMT) within a graft. Moreover, up-regulated expression of the CTGF gene is closely related to CAN [13].

This study aimed to determine the different effects of FK506 and MMF on the expression of CTGF and other fibrosis-associated genes in the kidney in rats that underwent CAN. We also wished to determine whether MMF can ameliorate the process of kidney allograft fibrosis in an experimental CAN rat model.

## Methods

### Animals

Male inbred Fischer (F344, RT1^lv1^) and Lewis (LEW, RT1^l^) rats weighing 250 to 300 g were obtained from Slac Laboratory Animals (Shanghai, China) and were fed with standard chow and water ad libitum. Fisher rats were kidney graft donors and Lewis rats were recipients. Animal experimentation was performed according to the National Research Council’s criteria (NIH No. 86–23) and Ethical Committee of the Guizhou Province People’s Hospital (No. 201102).

### Renal transplantation and experimental groups

Male inbred Fischer rat renal grafts were orthotopically transplanted into Lewis (LEW, RT1^l^) rats, as described previously [14]. In brief, the donor was anesthetized by intraperitoneal injection of urethane (1.0 mL/100 g body weight). The donor left kidneys were isolated and preserved in 0 °C to 4 °C heparin sodium chloride solution for 1 h to reinforce the cold ischemic injury before transplantation. After left native nephrectomy was performed in male Lewis recipient males, the donor renal artery were anastomosed to the aorta of the recipient using interrupted 9–0 nylon sutures. The donor vein was attached to the inferior vena of the recipient using the cuff technique. Right native nephrectomy was performed on the 10^th^ postoperative day. Allograft surgery which meets the following two criteria was considered as valid: (1) When vascular anastomosis is completed, the transplant kidneys immediately become red after circulation returns, with a certain degree of flexibility and hardness. There is no anastomotic bleeding, the renal artery beats well, and there is no renal vein distortion and no congestion. There is visible ureteral peristalsis and urine outflow in the ureteral orifice after 2–3 min. (2) The rats survive for more than 3 days after transplantation.

All recipient rats received Ciclosporin A (CsA) subcutaneously (10 mg/kg^-1^.d^-1^ × 10 d) from day 0 after transplantation (we used CsA for 10 days after transplantation to prevent the occurrence of acute rejection, to ensure that the FK506 and MMF groups were the same at baseline), and were then divided randomly into three oral treatment groups (each group, n = 9): (1) the vehicle group was given vehicle orally; (2) the FK506 group was given 0.15 mg/kg^-1^.d^-1^ FK506; and (3) the MMF group was given 20 mg/kg^-1^.d^-1^ MMF. The rats were sacrificed to harvest the renal allografts at 4, 8 and 12 weeks post-transplantation. Only kidneys without apparent complications of grafting were evaluated. Representative portions of the kidneys were snap-frozen in liquid nitrogen for nucleic acid and protein purification. Other samples were fixed in 4 % formalin or 70 % ethanol for histological evaluation. Blood was collected via direct heart punctuation for renal function assay.

### Renal function and histological evaluation

Serum creatinine (SCr) was measured using an automatic biochemistry analyzer. Kidneys were fixed in 4 % formaldehyde and 70 % ethanol for hematoxylin-eosin (HE) stain and periodic acid-Schiff (PAS) stain. The PAS reaction was used to assess the extent of glomerular, tubular and vascular obliteration. Chronic histological changes were graded from 0 to 3 according to Banff 97 criteria [3] (0, no lesions; 1, mild lesions; 2, moderate lesions; and 3, severe lesions). All graft kidney slides were assessed blindly by a pathologist.

### Quantitative real time PCR

Total RNA was extracted by using Trizol from tissue with the Total RNA Isolation kit (Invitrogen, USA). Total RNA was reverse transcribed using the PrimeScript RT reagent kit (Takara, Japan) according to the manufacturer’s instructions. CTGF was quantified by real-time PCR using SYBR Premix Ex Taq (TaKaRa, Japan) and the iCycleriQ sequence Detection System (Bio-Rad, USA). The primers used were as previously described [15]. The following cycling conditions were used: 95 °C for 10 min, 95 °C for 30 sec, 60 °C for 1 min, and 72 °C for 30 sec (40 cycles). The identity of PCR products was confirmed by sequencing. RT-PCR was performed in triplicate.

### Western blot

Total cell protein was extracted according to the manufacturer’s manual (M-PER Mammalian Protein Extraction Reagent) and transferred to a PVDF membrane. After blocking with PBS containing 5 % non-fat milk, the membrane was incubated with CTGF antibody (1:100 dilution, Santa Cruz, US), followed by incubation with HRP-labeled second antibody. GAPDH was used as the loading control.

### Immunohistochemistry

Fibrosis-associated genes, including alpha smooth muscle actin (α-SMA), E-cadherin, and collagen were detected and localized by immunohistology. In brief, 4-μm tissue sections were deparaffinized and antigen retrieval was performed. The sections were incubated with primary CTGF antibody (1:100), α-SMA antibody (1:100, Denmark), or E-cadherin (1:200 Santa Cruz, US), followed by incubation with HRP-labeled second antibody. After washing with PBS, the slides were then incubated with DAB for 10 min at room temperature and the results were observed under a microscope. The images were captured by Nikon spot cool CCD. The staining results were analyzed using image-Pro Plus multimedia color pathological image analysis system.

### Masson’s trichrome stain

Deparaffinized tissues sections were stained in Masson composition solution and light green SF solution. Collagen stains green, blood cells stain orange and muscle fibers stain red. The staining results were also analyzed using image-Pro Plus analysis system.

### Statistical analysis

SPSS11.0 software was used for analysis. The data are shown as mean ± SD. One-way analysis of variance (ANOVA) was used. Statistical significance was assessed by using a 2-tailed t-test and defined as p < 0.05.

## Results

### Renal function and histological evaluation

**Figure 1:**
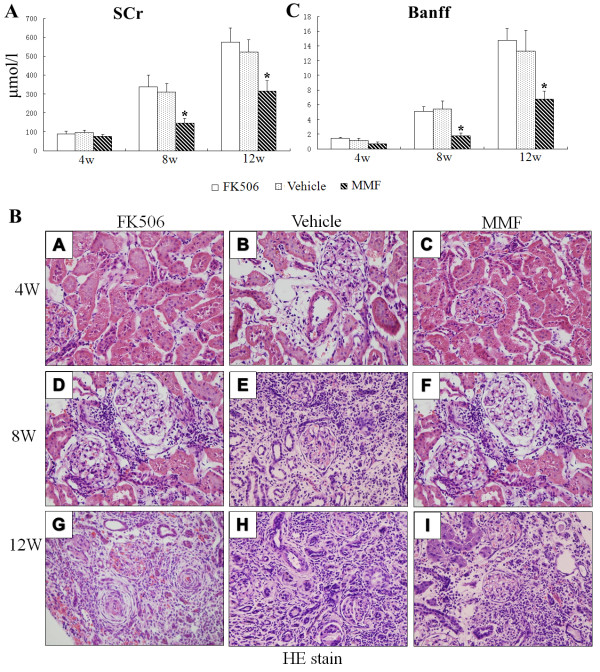
**Renal function and histopathological changes after transplantation.(A)** Serum creatinine levels were determined by automatic biochemistry analyzer 4, 8, and 12 weeks after transplantation. Values are shown as mean ± SD from three independent experiments. **(B)** HE staining was performed to determine histopathological changes. Original magnification: ×400. **(C)** The Banff score was scored by the quantitative criteria of Banff 97 using HE results. Values are shown as mean ± SD from three independent experiments. *Significant difference compared with the control group (p < 0.05).

The rat CAN model, which was reinforced through ischemic injury, was successfully achieved. SCr was assessed at 4, 8 and 12 weeks post-transplantation. All groups showed a continuous increase in SCr levels. However, these groups differed slightly from each other. SCr (mol/L) levels in the vehicle and Fk506 groups were remarkably increased (p < 0.05) at 8 weeks and 12 weeks post-transplantation compared with those in the MMF group (Figure 1A).

**Figure 2:**
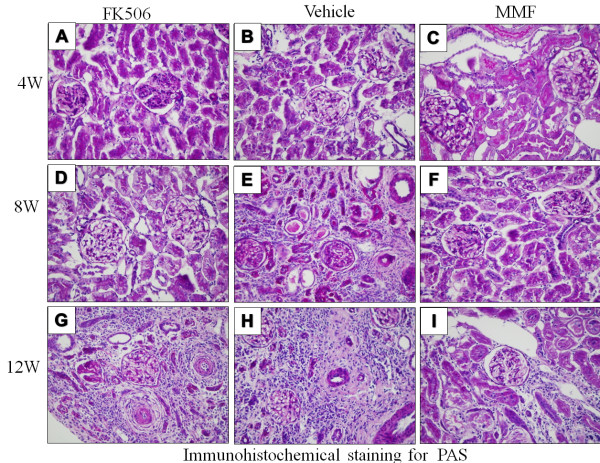
**Periodic acid-Schiff (PAS) stain.** The PAS reaction was used to assess the extent of glomerular, tubular and vascular obliteration.

Pathological changes of CAN progressively developed in rat kidney allografts. As shown in Figure. 1B and Figure 2, at 4 weeks post-transplantation, renal grafts presented with slight histological changes with only a few lymphocytes or mononuclear cell infiltration and without appearance of acute rejection. At 8 weeks post-transplantation, grafts showed various combinations of lesions, including tubular epithelial atrophy, interstitial fibrosis and arteriosclerosis. At 12 weeks, most of the tubules were damaged and there was severe interstitial fibrosis. Pathological lesions in the FK506 and vehicle groups were more severe than those in the MMF group. The Banff score in the MMF group was lower at 4 weeks compared with the vehicle and FK506 groups, but this was not significant (p > 0.05). However, at 8 weeks and 12 weeks post-transplantation, the Banff score of the vehicle and Fk506 groups was significantly higher than that of the MMF group (p < 0.05, Figure 1 C).

### MMF significantly inhibits CTGF expression in renal allografts

**Figure 3:**
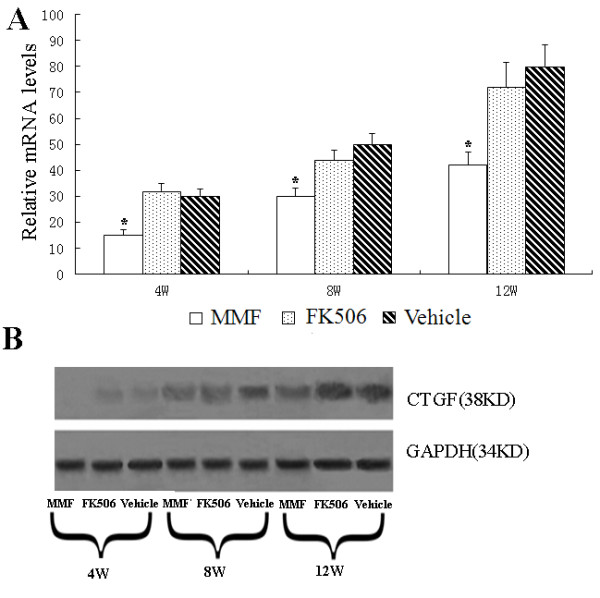
**Effect of MMF and FK506 on CTGF expression in renal allografts.(A)** Relative mRNA levels were determined by real-time PCR 4, 8, and 12 weeks after transplantation and normalized to the housekeeping gene GAPDH. Values are shown as mean ± SD from three independent experiments. *Significant difference compared with the control group (p < 0.05). **(B)** CTGF protein levels were determined by western blot and GAPDH was used as a housekeeping gene.

CTGF is a matricellular protein, which plays a crucial role in renal fibrosis. To determine whether CTGF was involved in the process of allograft fibrosis, we examined CTGF mRNA and protein expression levels in the kidney at 4, 8 and 12 weeks post-transplantation. Messenger RNA levels of CTGF in the MMF group were significantly lower at all three time points compared with those in the vehicle and FK506 groups (p < 0.05, Figure 3A). Protein expression of CTGF was negative in normal kidneys (data not shown) and began to be positively expressed in the vehicle and FK506 groups at 4 weeks post-transplantation. CTGF protein expression levels of all groups were still up-regulated at 8 weeks and 12 weeks. MMF treatment resulted in a significant decrease in CTGF expression compared with that in the other groups at 4, 8 and 12 weeks post-transplantation (p < 0.05, Figure 3B).

### The expression pattern of α-SMA, collagen and E-cadherin in renal allografts

Renal tubular epithelial cells (TEC) stimulated with physical or chemical factors, or induced by cytokines, can be converted into myofibroblast, which is an important event in fibrogenesis in renal grafts. Collagen, α-SMA, and E-cadherin are important molecular markers during the renal fibrosis process. To analyze the effect of the different immunosuppressive agents on renal fibrosis, we determined the expression of α-SMA, collagen deposition and E-cadherin in renal grafts by immunohistochemistry.

**Figure 4:**
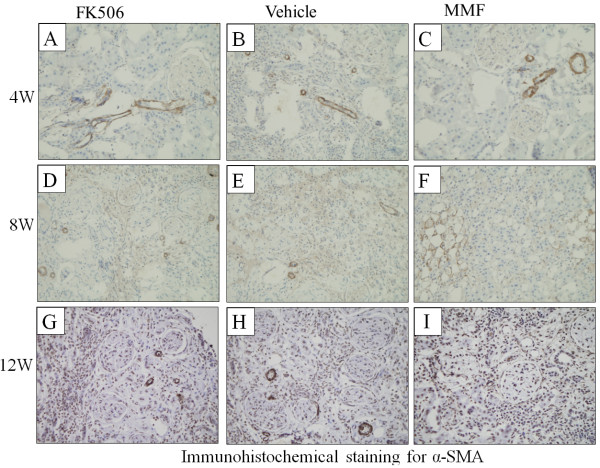
**Immunohistochemical staining for α-SMA expression.(B)**, **(E)**, and **(H)** show α-SMA expression of renal allografts in the vehicle group at postoperative days 4, 8, and 12, respectively. **(A)**, **(D)**, and **(G)** show α-SMA expression of renal allografts in the FK506 group at postoperative days 4, 8, and 12, respectively. **(C)**, **(F)**, and **(I)** show α-SMA expression of renal allografts in the MMF group at postoperative days 4, 8, and 12, respectively. Original magnification: ×400.

As shown in Figure 4, α-SMA, a molecular marker of myofibroblasts, was observed in TEC at 8 weeks and 12 weeks post-transplantation in all groups. The expression level of α-SMA in the MMF group was weaker compared with that in the vehicle and FK506 groups (p < 0.05) (Table 1).

**Table 1:** IOD values of α-SMA in each group 4, 8 and 12w post-transplantation (×10^4^)

	**Vehicle**	**FK506**	**MMF**
4w	4.6±0.3	4.8±0.3	4.3±0.4
8w	9.1±0.9	8.5±0.7	6.1±0.4*
12w	19.6±2.4	20.9±1.8	14.2±1.1*

*Significant difference compared with the vehicle group (*p<0.05*).

**Figure 5:**
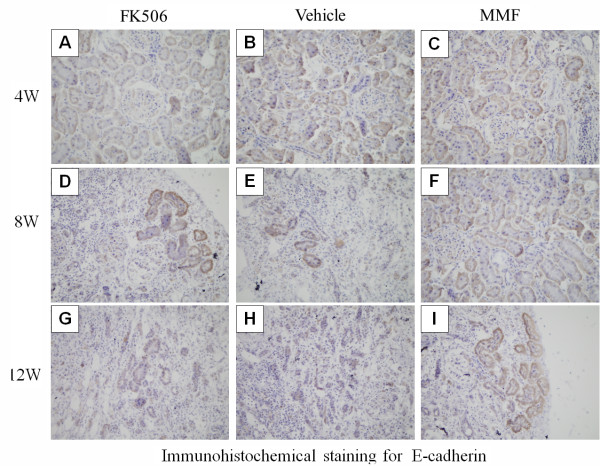
**Immunohistochemical staining for E-cadherin expression.(B)**, **(E)**, and **(H)** show E-cadherin expression of renal allografts in the vehicle group at postoperative days 4, 8, and 12, respectively. **(A)**, **(D)**, and **(G)** show E-cadherin expression of renal allografts in the FK506 group at postoperative days 4, 8, and 12, respectively. **(C)**, **(F)**, and **(I)** show E-cadherin expression of renal allografts in the MMF group at postoperative days 4, 8, and 12, respectively. Original magnification: ×400.

E-cadherin is a specific molecular marker of epithelium. E-cadherin expression was weaker in TEC in all groups at 8 weeks post-transplantation (Figure 5). However, at 12 weeks, atrophied tubules had lost E-cadherin and only a few of the remaining tubules were positive for E-cadherin. Moreover, E-cadherin expression in the MMF group was higher than that in the other groups at 4, 8 and 12 weeks post-transplantation (p < 0.05) (Table 2).

**Table 2:** IOD values of E-cadherin in each group at 4, 8 and 12w post-transplantation (×10^4^)

	**Vehicle**	**FK506**	**MMF**
4w	18.1±2.2	17.3±2.1	21.6±1.9
8w	7.6±0.6	7.0±0.9	13.5±1.2*
12w	4.2±0.9	4.9±0.6	9.1±1.1*

*Significant difference compared with the vehicle group (*p<0.05*).

**Figure 6:**
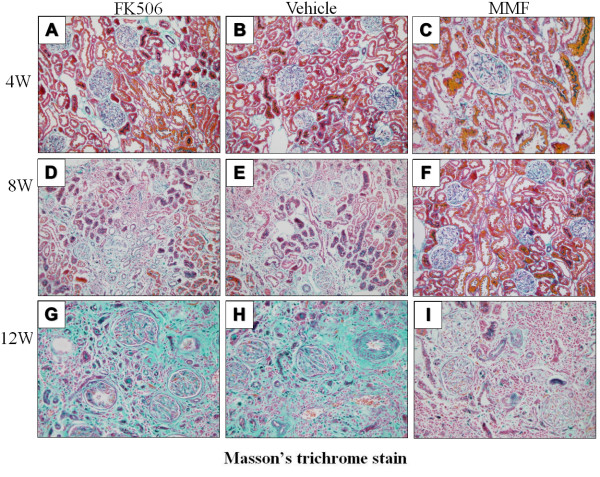
**Masson’s trichrome stain after transplantation.(B)**, **(E)**, and **(H)** show collagen deposition in the vehicle group at postoperative days 4, 8, and 12, respectively. **(A)**, **(D)**, and **(G)** show collagen deposition in the FK506 group at postoperative days 4, 8, and 12, respectively. **(C)**, **(F)**, and **(I)** show collagen deposition in the MMF group at postoperative days 4, 8, and 12, respectively. Original magnification: ×400.

Collagen deposition of renal grafts was detected by Masson’s trichrome stain. Collagen deposition was observed in the glomeruli and connective tissues around blood vessels, and it began to be positively expressed in the renal tubular basement membrane and kidney interstitium at 4 weeks post-transplantation. It continued to increase at 8 weeks and 12 weeks post-transplantation in all groups (Figure 6). Collagen deposition in the MMF group was significantly weaker compared with that in the vehicle and FK506 groups (p < 0.05) (Table 3).

**Table 3:** IOD values of Collagen deposition in each group at 4, 8 and 12w post-transplantation (×10^4^)

	**Vehicle**	**FK506**	**MMF**
4w	6.7±0.5	7.1±0.3	5.2±0.4*
8w	12.1±2.1	12.5±2.6	8.2±0.9*
12w	25.6±2.9	24.2±3.1	11.2±1.1*

*Significant difference compared with the vehicle group (*p<0.05*).

## Discussion

Like cyclosporine, FK506 is a calcineurin inhibitors (CNI) and binds to a corresponding protein FK506-binding protein 12 (FKBP12). These tacrolimus–immunophilin complexes inhibit the activity of calcineurin, which will impede calcium-dependent transduction and inactivate transcription factors, particularly the NFAT. NFAT is believed to initiate gene transcription for the formation of lymphokines such as interleukin-2 and interferon-c [16]. Thus, FK506 reduce acute rejection rates and improve patient and allograft survivals by interfering with T-cell activation. Nevertheless, CNI are toxic, and in particular, they are nephrotoxic. When strategies of reducing dose or withdrawal of FK506 with addition of MMF are implemented, there is a trend toward better kidney function [8]. Mycophenolatemofetil (MMF) is the first pharmaceutical prodrug of mycophenolic acid (MPA) and is used for preventing acute rejection. MPA inhibits the activity of inosine 5’-monophosphate dehydrogenase (IMPDH), the rate-limiting enzyme in the de novo pathway of guanosine nucleotides synthesis [17-19]. The pharmacological inhibition of IMPDH is considered to cause the depletion of guanosine nucleotides, leading to the suppression of cell proliferation in activated lymphocytes and a decrease of the adhesionmolecules expression [19,20]. This study aimed to determine its related mechanism through comparing the effect of FK506 and MMF on CAN as a single factor after transplantation. And we observed that MMF could inhibit renal fibrosis in an experimental animal CAN model by down-regulating the expression of TGF-β_1_ and Smad2 in grafts [10]. EMT is an important event in fibrogenesis of renal grafts, in which CTGF has been used potentially as a biomarker of EMT [11]. In the present study, we evaluated the expression of CTGF and fibrosis-associated genes in CAN rats treated with FK-506 or MMF to determine whether there are differences between these compounds. The pathological profile demonstrated that CAN lesions were milder in the MMF group than those in the vehicle and FK506 groups, and MMF could effectively prevent fibrosis in kidney allografts, possibly by reducing the expression of CTGF, α-SMA and collagen. In addition, although there was obvious histopathological damage in the MMF group at 12 week, HE and Masson’s trichrome staining results showed some parts of glomeruli remain functional. Since Scr levels significantly increased only when glomerular filtration rate was reduced to 1/3 of normal levels, it maintains a significant low level in MMF group compared with other groups at 12 week.

Renal fibrosis correlated with renal TEC lose their polarized phenotype and acquire new characteristic features of myofibroblasts, the major effector cells responsible for the excess deposition of interstitial extracellular matrixc (ECM) under pathological conditions [21]. Alpha-SMA is a specific molecular marker of activated myofibroblasts, and a high expression level of α-SMA is associated with the differentiation from epithelial cells to myofibroblasts [22,23]. E-cadherin is an adhesive junction protein expressed in differentiated and polarized epithelial cells [24]. Under pathological conditions, TEC lose E-cadherin. The disappearance of E-cadherin in epithelial cells indicates that the cell undergoes an EMT process [25]. Both α-SMA and E-cadherin are extracellular matrix proteins correlated with the renal fibrosis process [26,27]. Once secreted by ECM producing cells, collagen accumulates in the basement or interstitial space. Disordered synthesis and secretion and unsatisfactory degradation leads to the excessive deposition of ECM components and destruction of tissue architecture. In this study, we evaluated fibrosis-associated genes to analyze whether FK506 and MMF modulate the renal fibrosis process in a different manner. Our results showed that FK506 significantly up-regulated the expression of α-SMA and collagen, and down-regulated the expression of E-cadherin in rat kidney grafts. MMF reduced these effects, suggesting that MMF could effectively attenuate the fibrosis process in renal allografts by decreasing the expression of fibrosis-associated genes.

In this study, we also noticed that there was no significant difference in the level of CAN and renal fibrosis between FK506 and vehicle groups. In this study, we used an enhanced ischemic model which we established previously, it could successfully accelerate the development and progression of CAN in the early stage. We used CsA to prevent AR after kidney transplantation. And after that, vehicle group did not use immunosuppressive agents and therefore, the rejection may accelerate the development of CAN and renal fibrosis. To some extent, the use of FK506 could suppress immune rejection, but its renal toxicity had also accelerated the development of CAN and renal fibrosis. Although there was difference between these two groups in the progression of CAN and renal fibrosis, however, with no statistical significant difference. In addition, the combination of MMF and FK506 is often used in clinical kidney transplantations, further studies are needed to clarify the combined effects.

CTGF is one of the key cytokines in renal fibrosis, and it plays an important role in EMT of TEC. Our study demonstrated that MMF, but not FK506, down-regulated the expression of CTGF in rat kidney allografts. A correlation between MMF and EMT might provide an explanation of the mechanism of action of MMF to ameliorate transplant fibrosis in experimental models of CAN. The above results suggested that fibrosis-associated genes, especially CTGF, are involved in the compatibility of MMF to ameliorate the process of renal fibrosis within an experimental model of CAN. Furthermore, our previous results also showed that the change in CTGF expression was earlier than the pathological changes (data not shown). Therefore, CTGF has the potential to be a biomarker of CAN, as well as a therapeutic target in the management of graft fibrosis [13].

## Conclusions

In summary, our study confirms that MMF can significantly improve graft function and structure, and attenuate the expression of fibrosis-associated genes. This suggests that MMF may inhibit the progress of EMT in rat CAN and block fibrosis in rats undergoing CAN. FK506 can facilitate the development of renal fibrosis in rat CAN.

## Abbreviations

FK506, Tacrolimus; MMF, Mycophenolatemofetil; CsA, Ciclosporin A; CAN, Chronic allograft nephropathy; SCr, Serum creatinine; CTGF, Connective tissue growth factor; EMT, Epithelial-mesenchymal transformation; TEC, Tubular epithelial cells; CNI, Calcineurin inhibitor; α-SMA, Alpha smooth muscle actin; HE, Hematoxylin-eosin; PAS, Periodic acid-schiff; ECM, Extracellular matrix.

## Competing interests

The authors have declared no conflict of interest.

## Authors’ contributions

LL and GHL conceived the study, established the design and carried out the experimental work. ZLS and WDW participated in the data analysis and provided critical comments on the study design and manuscript. LL drafted this manuscript. All authors read and approved the final manuscript.

## Authors’ information

Lei Luo and Zhaolin Sun are co-authors.

## Pre-publication history

The pre-publication history for this paper can be accessed here:

http://www.biomedcentral.com/1471-2369/13/53/prepub

## References

[B1] Chapman JR O’Connell PJ Nankivell BJ Chronic renal allograft dysfunction J Am SocNephrol 2005 16 3015 26 10.1681/ASN.2005050463 16120819

[B2] Joosten SA van Kooten C Sijpkens YW deFijter JW Paul LC The pathobiology of chronic allograft nephropathy: immune-mediated damage and accelerated aging Kidney Int 2004 65 1556 9 10.1111/j.1523-1755.2004.05410.x 15086891

[B3] Racusen LC Solez K Colvin RB Bonsib SM Castro MC Cavallo T The Banff 97 working classification of renal allograft pathology Kidney Int 1999 55 713 23 10.1046/j.1523-1755.1999.00299.x 9987096

[B4] Nankivell BJ Borrows RJ Fung CL O’Connell PJ Allen RD Chapman JR The natural history of chronic allograft nephropathy N Engl J Med 2003 349 2326 33 10.1056/NEJMoa020009 14668458

[B5] Campistol JM Boletis IN Dantal J de Fijter JW Hertig A Neumayer HH Chronic allograft nephropathy – a clinical syndrome: early detection and the potential role of proliferation signal inhibitors Clin Transplant 2009 23 769 777 10.1111/j.1399-0012.2009.01057.x 19719730

[B6] Shihab FS Bennett WM Tanner AM Andoh TF Mechanism of fibrosis in experimental tacrolimus nephrotoxicity Transplantation 1997 64 12 1829 10.1097/00007890-199712270-00034 9422427

[B7] Randhawa PS Shapiro R Jordan ML Starzl TE Demetris AJ The histopathological changes associated with allograft rejection and drug toxicity in renal transplant recipients maintained on FK 506: Clinical significance and comparison with cyclosporine Am J SurgPathol 1993 17 1 60 10.1097/00000478-199301000-00007 PMC3229279 7680544

[B8] Lo A Egidi MF Gaber LW Amiri HS Vera S Nezakatgoo N Comparison of sirolimus-based calcineurin inhibitor-sparing and calcineurin inhibitor free regimens in cadaveric renal transplantation Transplantation 2004 77 8 1228 1235 10.1097/01.TP.0000121504.69676.5E 15114090

[B9] Kaminska D Tyran B Mazanowska O Letachowicz W Kochman A Rabczynski J Mycophenolate Mofetil but Not the Type of Calcineurin Inhibitor (Cyclosporine vsTacrolimus) Influences the Intragraft mRNA Expression of Cytokines in Human Kidney Allograft Biopsies byIn Situ RT-PCR Analysis Transplant Proc 2005 37 2 770 772 10.1016/j.transproceed.2004.12.144 15848526

[B10] Gao R Lu Y Xin YP Zhang XH Wang J Li YP The effects of different immunosuppressants on chronic allograft nephropathy by affecting the transforming growth factor-beta and Smads signal pathways Transplant Proc 2006 38 7 2154 2157 10.1016/j.transproceed.2006.06.006 16980029

[B11] Teng D Lu YP Gao R Xin Y Cao G Li X Conversion From Cyclosporine to Mycophenolate Mofetil Improves Expression of A20 in the Rat Kidney Allografts Undergoing Chronic Rejection Transplant Proc 2006 38 7 2164 2167 10.1016/j.transproceed.2006.06.007 16980032

[B12] Leask A Transcriptional profiling of the scleroderma fibroblast reveals a potential role for connective tissue growth factor (CTGF) in pathological fibrosis Keio J Med 2004 53 2 74 7 10.2302/kjm.53.74 15247510

[B13] Cheng O Thuillier R Sampson E Schultz G Ruiz P Zhang X Connective tissue growth factor is a biomarker and mediator of kidney allograft fibrosis Am J Transplant 2006 6 10 2292 2306 10.1111/j.1600-6143.2006.01493.x 16889607

[B14] Lu YP Chen WG Wang I Li YP A new rat model of transplant arteriosclerosis accelerated by I/R injury Transplant Proc 2003 35 184 10.1016/S0041-1345(02)03942-8 12591358

[B15] Luo GH Lu YP Song J Yang L Shi YJ Li YP Inhibition of connection tissue growth factor by small interfering RNA prevents renal fibrosis in rats undergoing chronic allograft nephropathy Transplantation Proc 2008 40 2365 2369 10.1016/j.transproceed.2008.07.100 18790236

[B16] Gummert JF Ikonen T Morris RE Newer immunosuppressive drugs: A review J Am SocNephrol 1999 10 1366 1380 10.1681/ASN.V1061366 10361877

[B17] Jackson RC Weber G Morris HP IMP dehydrogenase, an enzyme linked with proliferation and malignancy Nature 1975 256 331 3 10.1038/256331a0 167289

[B18] Sintchak MD Nimmesgern E The structure of inosine 5'-monophosphate dehydrogenase and the design of novel inhibitors Immunopharmacology 2000 47 163 84 10.1016/S0162-3109(00)00193-4 10878288

[B19] Allison AC Eugui EM Mycophenolatemofetil and its mechanisms of action Immunopharmacology 2000 47 85 118 10.1016/S0162-3109(00)00188-0 10878285

[B20] Eugui EM Almquist SJ Muller CD Allison AC Lymphocyte-selective cytostatic and immunosuppressive effects of mycophenolic acid in vitro: role of deoxyguanosine nucleotide depletion Scand J Immunol 1991 33 161 73 10.1111/j.1365-3083.1991.tb03746.x 1826793

[B21] Zeisberg M Kalluri R The role of epithelial-to-mesenchymal transition in renal fibrosis J Mol Med 2004 82 3 175 81 10.1007/s00109-003-0517-9 14752606

[B22] Thannickal VJ Lee DY White ES Cui Z Larios JM Chacon R Myofibroblast differentiation by transforming growth factor-beta1 is dependent on cell adhesion and integrin signaling via focal adhesion kinase J BiolChem 2003 278 14 12384 9 10.1074/jbc.M208544200 12531888

[B23] Badid C Desmouliere A Babici D Hadj-Aissa A McGregor B Lefrancois N Interstitial expression of alpha-SMA: an early marker of chronic renal allograft dysfunction Nephrol Dial Transplant 2002 17 11 1993 8 10.1093/ndt/17.11.1993 12401859

[B24] Medici D Hay ED Goodenough DA Cooperation between snail and LEF-1 transcription factors is essential for TGF-beta1-induced epithelial-mesenchymal transition MolBiol Cell 2006 17 4 1871 9 10.1091/mbc.E05-08-0767 PMC1415320 16467384

[B25] Vongwiwatana A Tasanarong A Rayner DC Melk A Halloran PF Epithelial to mesenchymal transition during late deterioration of human kidney transplants: the role of tubular cells in fibrogenesis Am J Transplant 2005 5 6 1367 74 10.1111/j.1600-6143.2005.00843.x 15888043

[B26] Ortega-Velazquez R Gonzalez-Rubio M Ruiz-Torres MP Diez-Margues ML Lglesias MC Rodriguez-Puyol M Collagen I upregulates extracellular matrix gene expression and secretion of TGF-beta 1 by cultured human mesangial cells Am J Cell Physiol 2004 286 6 C1335 43 10.1152/ajpcell.00279.2003 14761892

[B27] Lam S van der Geest RN Verhagen NA Daha MR van Kooten C Secretion of collagen type IV by human renal fibroblasts is increased by high glucose via a TGF-beta- independent pathway Nephrol Dial Transplant 2004 19 7 1694 701 10.1093/ndt/gfh235 15150349

